# Supplementary material for: Tumor-Associated Macrophage (TAM)-Related Cytokines, sCD163, CCL2, and CCL4, as Novel Biomarkers for Overall Survival and Time to Treatment in Waldenstrom’s Macroglobulinemia: Emphasis on Asymptomatic WM
Source: Cells. 2025 Feb 13;14(4):275. doi: 10.3390/cells14040275 (PMC11853255; doi:10.3390/cells14040275)
Supplement: Supplementary file 1 [file cells-14-00275-s001.zip › cells-3455587-supplementary.pdf]

Supplementary Material

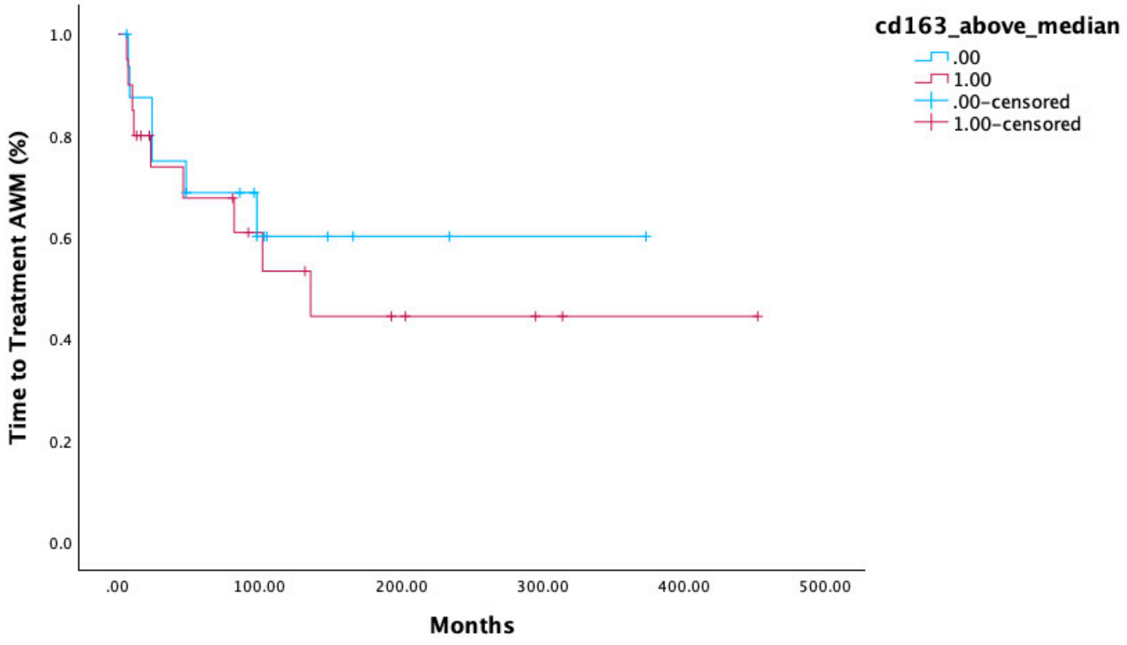

Figure S1: *TTT in AWM patients with sCD163 above the median (p = 0.544).*

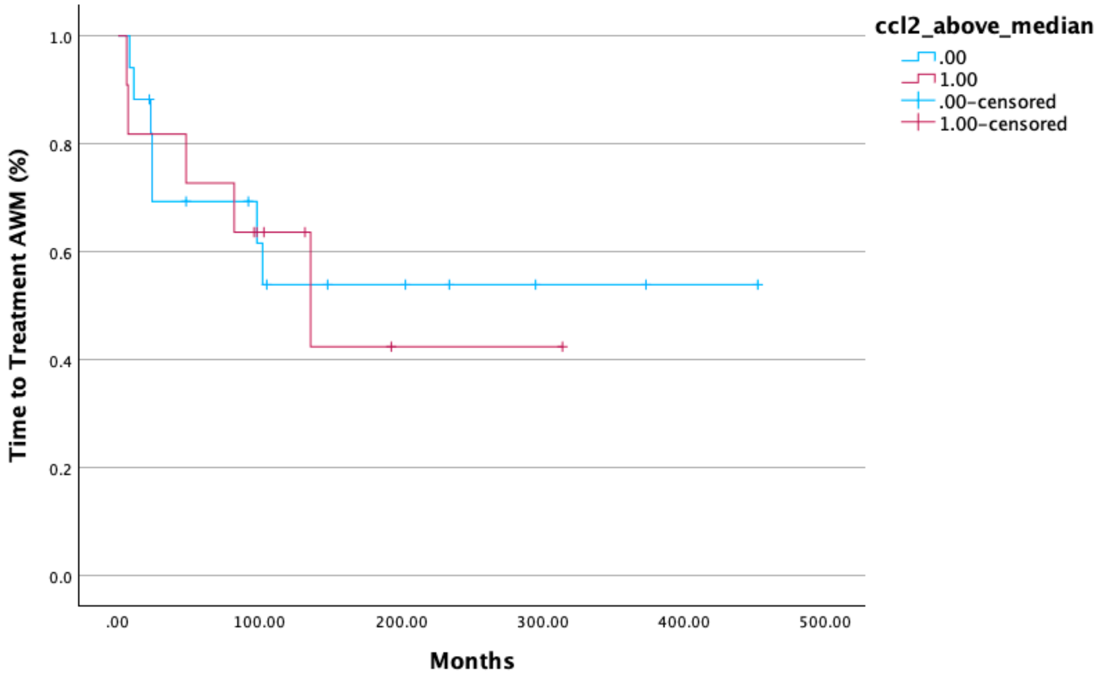

Figure S2: *TTT in AWM patients with CCL2 above the median (p = 0.854).*

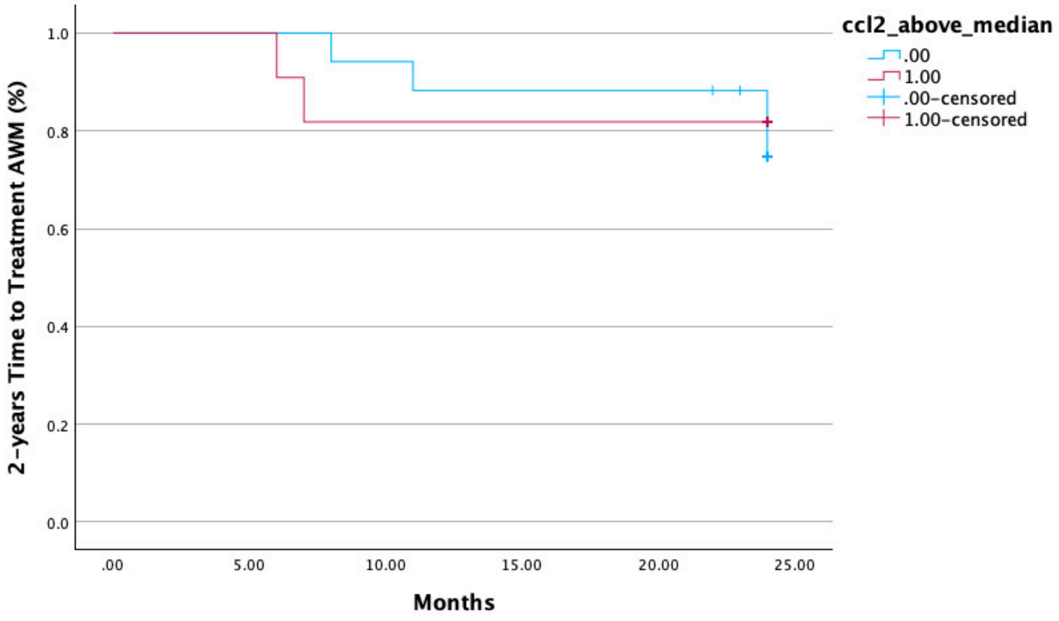

Figure S3: *2-years TTT in AWM patients with CCL2 above the median (p = 0.807).*

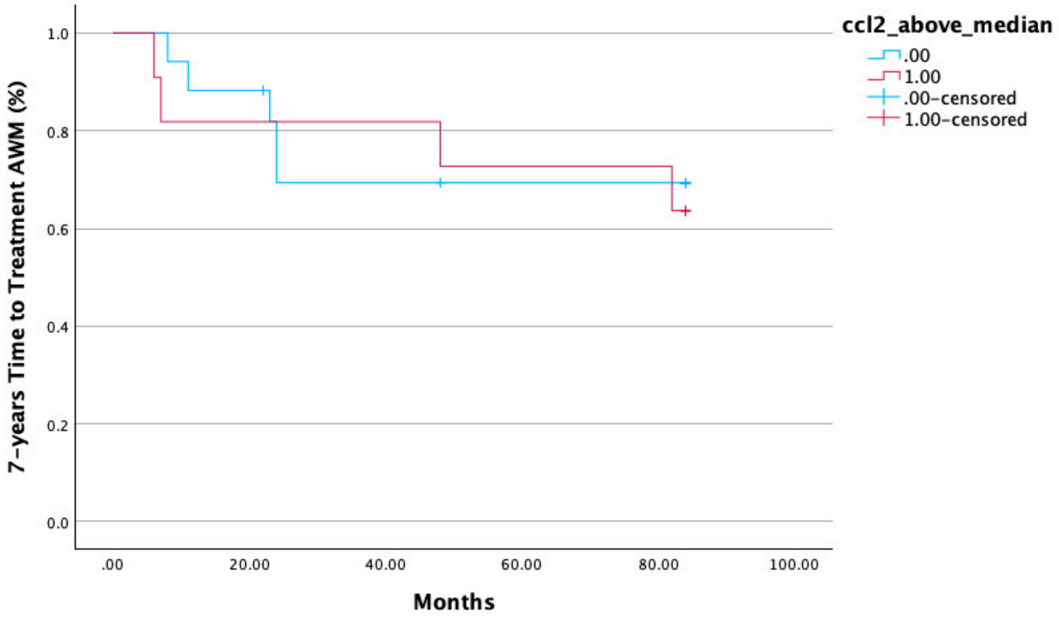

Figure S4: *7-years TTT in AWM patients with CCL2 above the median (p = 0.77).*

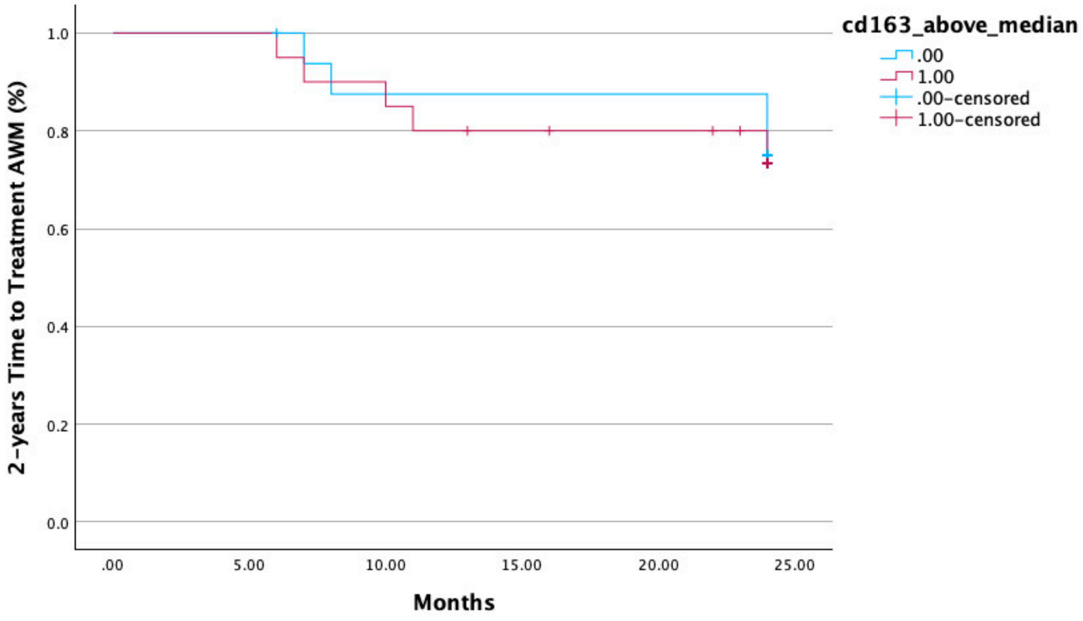

Figure S5: *2-years TTT in AWM patients with sCD163 above the median (p = 0.821).*

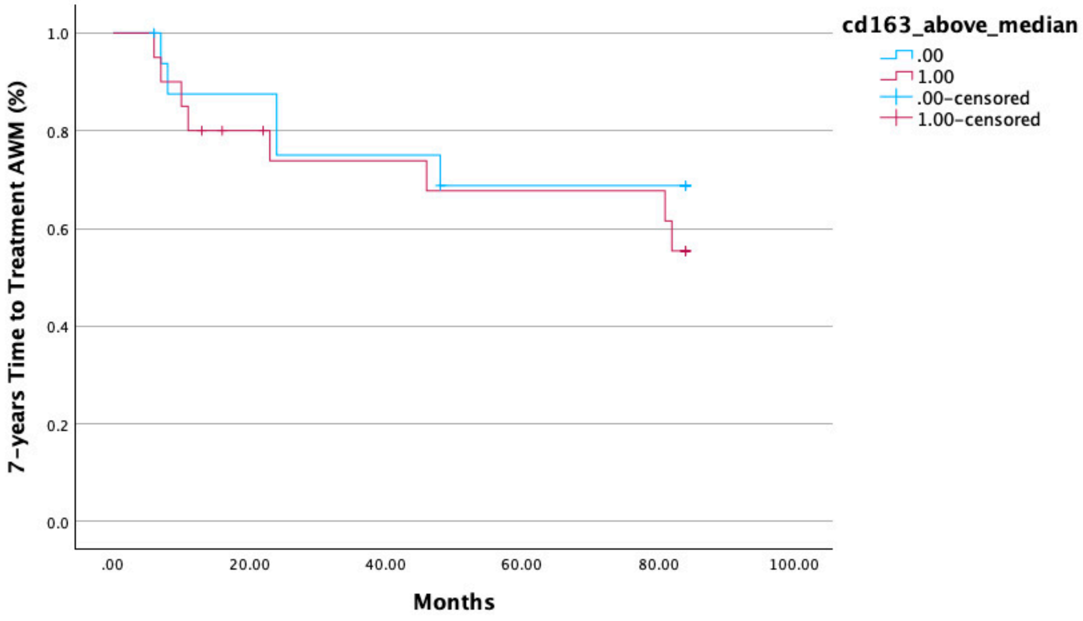

Figure S6: *7-years TTT in AWM patients with sCD163 above the median (p = 0.464).*

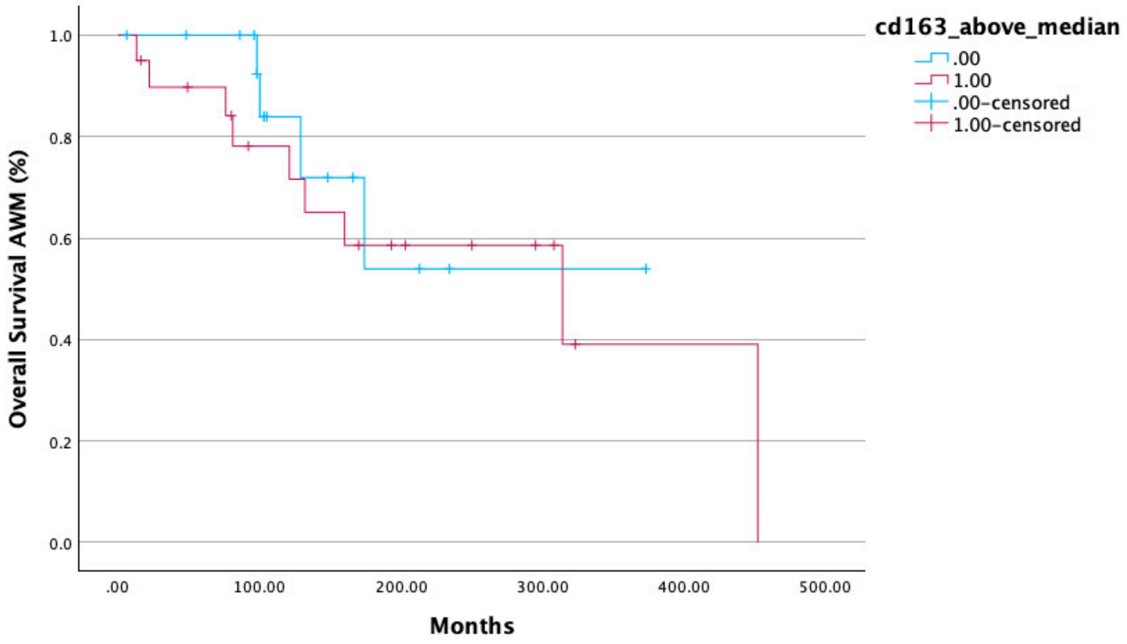

Figure S7: *OS in all WM patients with a values of sCD163 above the median (p = 0.575).*

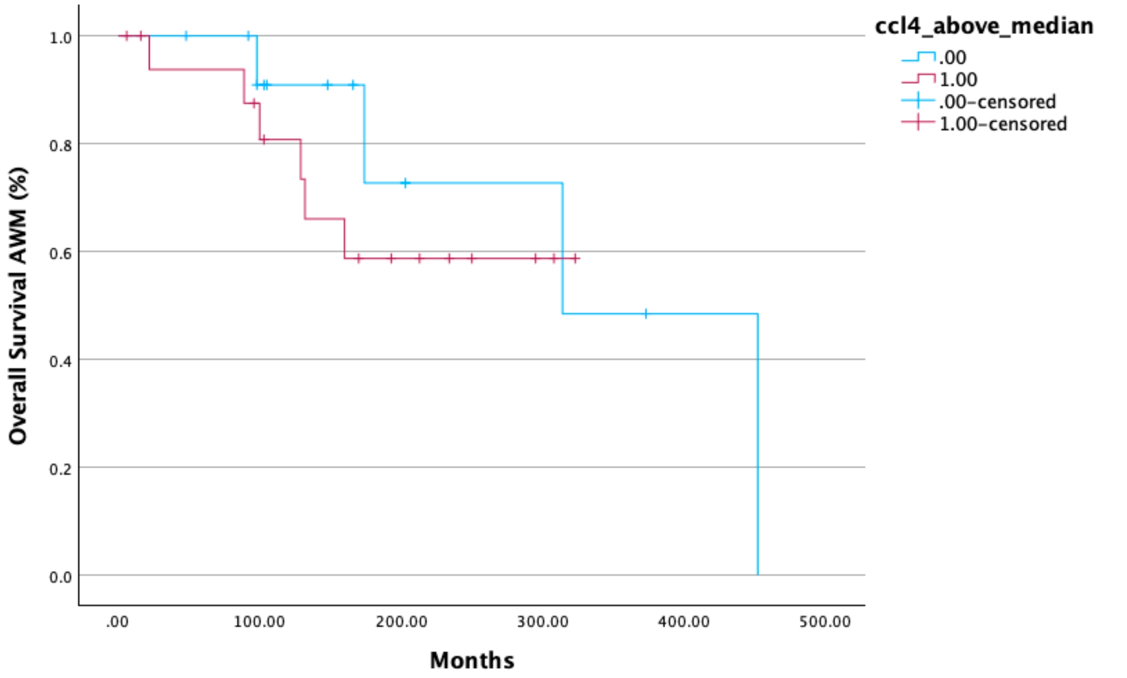

Figure S8: *OS in all WM patients with a values of CCL4 above the median (p = 0.44).*
